# Clinical and sociodemographic factors associated with the quality of life of children and adolescents with type 1 diabetes*

**DOI:** 10.1590/1980-220X-REEUSP-2023-0195en

**Published:** 2024-01-19

**Authors:** Elisabeth Luisa Rodrigues Ramalho, Valéria de Cássia Sparapani, Rebecca Ortiz La Banca Barber, Renata Cardoso Oliveira, Lucila Castanheira Nascimento, Neusa Collet

**Affiliations:** 1 Universidade Federal de Pernambuco, Recife, PE, Brazil.; 2 Universidade Federal de Santa Catarina, Departamento de Enfermagem, Florianópolis, SC, Brazil.; 3 Institute for Nursing and Interprofessional Research, Children’s Hospital Los Angeles, Los Angeles, EUA.; 4 Universidade Federal da Paraíba, João Pessoa, PB, Brazil.; 5 Universidade de São Paulo, Escola de Enfermagem de Ribeirão Preto, Departamento de Enfermagem Materno-Infantil e Saúde Pública, Ribeirão Preto, SP, Brazil.; 6 Universidade Federal da Paraíba, Departamento de Enfermagem de Saúde Coletiva, João Pessoa, PB, Brazil.

**Keywords:** Diabetes Mellitus, Type 1, Child, Adolescent, Quality of Life, Health Profile, Diabetes Mellitus Tipo 1, Niño, Adolescente, Calidad de Vida, Perfil de Salud, Diabetes Mellitus Tipo 1, Criança, Adolescente, Qualidade de Vida, Perfil de Saúde

## Abstract

**Objective:**

To analyze clinical and sociodemographic factors associated with the health-related quality of life of children and adolescents with type 1 Diabetes Mellitus.

**Method:**

A quantitative, cross-sectional and analytical study, developed in a municipality in northeastern Brazil, between March and September 2021, with 81 children/adolescents with type 1 Diabetes Mellitus and their guardians/caregivers. A questionnaire containing sociodemographic and clinical variables and two quality of life instruments were used. Descriptive and inferential analysis was carried out.

**Results:**

Adolescents whose parents had a family income greater than a minimum wage had a lower prevalence of impaired quality of life when compared to those with a lower income. Adolescents with time since diagnosis of less than four years had a satisfactory quality of life, and children aged 8 to 12 years who self-administered insulin had a lower prevalence of high quality of life compared to those who did not.

**Conclusion:**

Adolescents with a family income of less than a minimum wage, diagnosis time of more than four years and children aged 8–12 who self-administer insulin need greater professional support to have a better quality of life.

## RESUMO

**Objetivo:**

Analisar os fatores clínicos e sociodemográficos associados à qualidade de vida relacionada à saúde de crianças e adolescentes com Diabetes Mellitus tipo 1.

**Método:**

Estudo quantitativo, transversal e analítico, desenvolvido em um município do Nordeste brasileiro, entre março e setembro de 2021, com 81 crianças/adolescentes com Diabetes Mellitus tipo 1 e seus responsáveis/cuidadores. Utilizaram-se um questionário contendo as variáveis sociodemográficas e clínicas e dois instrumentos de qualidade de vida. Realizou-se análise descritiva e inferencial.

**Resultados:**

Adolescentes cujos pais tinham renda familiar superior a um salário mínimo apresentaram prevalência menor de qualidade de vida prejudicada, quando comparados àqueles com renda inferior. Adolescentes com tempo de diagnóstico menor que quatro anos apresentaram qualidade de vida satisfatória, e crianças de 8 a 12 anos que faziam autoaplicação de insulina apresentaram prevalência menor de qualidade de vida alta em relação às que não realizavam.

**Conclusão:**

Adolescentes com renda familiar inferior a um salário mínimo, tempo de diagnóstico superior a quatro anos e crianças de 8–12 anos que realizam autoaplicação de insulina necessitam de maior suporte profissional para ter melhor qualidade de vida.

## RESUMEN

**Objetivo:**

Analizar los factores clínicos y sociodemográficos asociados a la calidad de vida relacionada con la salud de niños y adolescentes con Diabetes Mellitus tipo 1.

**Método:**

Estudio cuantitativo, transversal y analítico, desarrollado en un municipio del Nordeste brasileño, entre marzo y septiembre de 2021, con 81 niños/adolescentes con Diabetes Mellitus tipo 1 y sus tutores/cuidadores. Se utilizó un cuestionario que contenía variables sociodemográficas y clínicas y dos instrumentos de calidad de vida. Se realizó análisis descriptivo e inferencial.

**Resultados:**

los adolescentes cuyos padres tenían un ingreso familiar superior a un salario mínimo tuvieron una menor prevalencia de deterioro de la calidad de vida en comparación con aquellos con menores ingresos. Los adolescentes con un tiempo de diagnóstico menor a cuatro años tuvieron una calidad de vida satisfactoria, y los niños de 8 a 12 años que se autoadministraron insulina tuvieron una menor prevalencia de alta calidad de vida en comparación con los que no lo hicieron.

**Conclusión:**

Los adolescentes con ingreso familiar menor a un salario mínimo, tiempo de diagnóstico mayor a cuatro años y niños de 8 a 12 años que se autoadministran insulina necesitan mayor apoyo profesional para tener una mejor calidad de vida.

## INTRODUCTION

Type 1 Diabetes Mellitus (1DM) is considered one of the most prevalent endocrine-metabolic diseases in childhood and adolescence^(1)^. It presents severe insulin deficiency due to the destruction of ß cells associated with autoimmunity. Clinical presentation is abrupt, with a propensity for ketosis and ketoacidosis, requiring full insulin therapy from diagnosis or after a short period^(2, 3, 4, 5)^. It is a complex disease that requires continuous health care^(1)^ throughout life.

The incidence of 1DM has increased substantially in recent decades, and continues to grow in global proportions, in addition to already affecting approximately 1.52 million individuals in the age group from 0 to 20 years^(6)^. In the ranking of countries with the highest number of children/adolescents with 1DM, Brazil ranks third, which represents a serious public health concern at national level^(1,6)^.

Experiencing 1DM in childhood or adolescence is a challenging experience that generates conflicts and difficulties given the unpredictability of the disease due to demands and changes in lifestyle required by treatment. From the moment of diagnosis, care can become complex as it includes multiple daily tasks that impact family dynamics, in addition to obstacles in incorporating therapy due to clinical and sociodemographic factors that can interfere with children’s and adolescents’ health status^(7)^. It is recognized that these factors interfere with individuals’ management of their disease and can result in overload and a consequent high level of stress, significantly impacting their quality of life (QoL)^(8)^.

QoL, based on the World Health Organization, refers to “an individual’s perception of their position in life in the context of the culture and value systems in which they live and in relation to their goals, expectations, standards and concerns”^(9)^. The definition of QoL goes far beyond the effects of the disease on individuals’ functional status, as it also involves the importance of cultural meaning systems, values and personal preferences. This construct encompasses psychosocial adjustment, well-being, self-esteem, stress and coping strategies^(10)^, which justifies carrying out studies that investigate the gaps regarding this topic.

Health-related quality of life (HRQoL) reflects the intention of quantifying the repercussions of an illness and its treatment, according to the perception that people have about their ability to develop their potential and lead a full life. HRQoL is related to the perception that individuals have of both the impact of their dysfunction and its existence^(11)^.

The assessment of HRQoL of children and adolescents with 1DM allows health professionals to assist in formulating appropriate strategies and behaviors that motivate them to self-care and minimize complications arising from the disease^(4)^. It is noteworthy that data regarding the clinical, sociodemographic and QoL profile for this phase of human development are still little explored nationally.

Considering the above, the following research question was created: what are the associations between QoL levels and clinical and sociodemographic profile of children and adolescents with 1DM? Therefore, the study aimed to analyze clinical and sociodemographic factors associated with the HRQoL of children and adolescents with 1DM.

## METHOD

### Study Design

This is a quantitative, cross-sectional and analytical study, linked to a macro project and guided by STrengthening the Reporting of OBservational studies in Epidemiology (STROBE).

### Site

The study was carried out in two reference centers for the treatment and monitoring of children and adolescents with 1DM, in a large city in Paraíba in northeastern Brazil.

### Population

The study population consisted of children and adolescents diagnosed with 1DM treated at two reference centers specializing in endocrinological diseases. In these centers, children and adolescents with 1DM are treated on a quarterly basis at the service’s outpatient clinics, which are scheduled according to metabolic control and identified needs. The mean number of consultations was 10 visits per week, and approximately 101 children and adolescents with 1DM were registered in the health services chosen to carry out the research. Recruitment occurred through a list made available by the medical and nursing team with information about the diagnosis, in addition to periodic visits by researchers to health services.

### Selection Criteria

Eligibility criteria were established as children and adolescents from two to 18 years old, with a diagnosis of 1DM for at least six months, symptomatic or using insulin since diagnosis and continuously afterwards, with a history of hyperglycemia consistent with diabetes in the absence of laboratory tests at diagnosis that showed hyperglycemia documented by one of the following tests: fasting blood glucose ≥ 126 mg/dl; oral glucose tolerance test (OGTT) ≥ 200 mg/dl; HbA1c ≥ 6.5%; capillary blood glucose ≥ 200 mg/dl^(12)^. In addition to this audience, their legal guardians were also invited to participate in the research, but those who were illiterate were not included, as HRQoL instruments were self-administered. Children and adolescents who had a record of care, but who failed to undergo follow-up at the service for more than a year, were excluded.

### Sample Definition

The sample was non-probabilistic for convenience. Contact with participants occurred in person and/or remotely, depending on their preference. Of the 101 individuals, 16 did not participate due to not showing up at the outpatient clinic on the day of the previously scheduled medical appointment as well as for the application of research instruments, or for not answering the researchers’ calls on the date of the remote interview, previously scheduled via messaging application. Among the remaining 85 participants, four guardians refused to participate due to unavailability and/or fear of disclosing the requested information, despite the guarantee of anonymity. The sample of this study consisted of 81 children/adolescents with 1DM and their respective legal guardians.

### Data Collection

**Figure 1:**
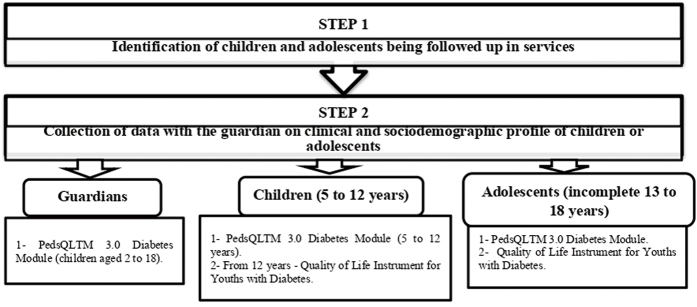
Data collection flowchart.

Data collection took place in a hybrid way, in person and/or remotely, through telephone calls and use of messaging application, between March and September 2021, in two steps, as shown in Figure 1. It was carried out by two previously trained researchers and qualified.

Three instruments were used to collect data. The first was the form containing 76 questions related to clinical and sociodemographic variables (sex, age, education, time since diagnosis, glycated hemoglobin, insulin therapy, capillary blood glucose monitoring, nutritional aspects, physical activity, acute and long-term complications), prepared by the multicenter diabetes group based on the collaboration of researchers and educators in the area based on guidelines^(2)^. This instrument was applied to the main caregiver, and was completed by the researcher in charge. The second was the QoL instrument Pediatric Quality of Life Inventory^TM^ 3.0 Diabetes Module (PedsQLTM 3.0 Diabetes Module), composed of 23 items and four domains: diabetes problems (diabetes symptoms); problems with treatment I (barriers to treatment); problems with treatment II (adherence to treatment); and concerns and problems with communication. All questions are Likert-type, and have five answer options, ranging from “never a problem” (score 0) to “almost always a problem” (score 4). The total and domain scores are calculated by adding them together, with higher scores indicating better HRQoL. This instrument can be applied both to children and adolescents in the age group from 2 to 18 years of age and to their parents. Total score analysis was 0.88 for children and parents according to Cronbach’s alpha coefficient, which indicates excellent reliability in internal consistency^(13)^.

Finally, the Quality of Life Instrument for Youths with Diabetes (IQVJD – *Instrumento de Qualidade de Vida de Jovens com Diabetes*) was used, originally created in English and adapted to Brazilian culture in 2004 by researcher Novato^(14)^. The IQVJD consists of 50 items, with questions subdivided into three domains: satisfaction (17 questions); impact (22 questions); and concern (11 questions). All questions are Likert-type, and have five answer options, which vary between “very satisfied” (score 1) and “very dissatisfied” (score 5) in the satisfaction domain, and between “never” (score 1) and “always” (score 5) in the impact and concerns areas. In addition to these, there is a question that comprises adolescents’ self-perception in relation to their health, whose answers vary between “excellent”, “good”, “satisfactory” and “bad”. This instrument is self-administered and aimed at adolescents in the age group aged 12 to 18 years. The total and per domain scores are calculated by adding them together, with the lowest score suggesting better HRQoL, and the highest, worse HRQoL, except for item B7 of the impact domain, which is inverted^(12)^. Application of the form and QoL instruments took approximately 40 minutes.

### Data Analysis and Treatment

Data were organized in a Microsoft Office Excel^®^ spreadsheet through double entry and subsequent validation, in order to control possible errors in transposing the information. They were then submitted to the Statistical Package for the Social Sciences (SPSS^®^) version 18.0 for the descriptive statistical analysis of sample characterization, which occurred with the calculation of absolute and relative frequencies, measures of central tendency (mean, median) and variability (standard deviations and interquartiles). Regarding numeric variables, the Shapiro-Wilk and Kolmogorov-Smirnov tests were applied in order to verify adherence to normality, i.e., the distribution of these data.

In the bivariate analysis, non-parametric Pearson’s chi-square and Fisher’s exact tests were used to verify associations between QoL scores (dependent variable) and sociodemographic and clinical characteristics (independent variables). The epidemiological association measure adopted was the Prevalence Ratio (PR), being considered significant when p ≤ 0.05 (5% significance level), with 95% Confidence Intervals.

It should be noted that, in the inferential analysis, a statistical cutoff point was used for the IQVJD and PedsQl instruments based on quartiles. In PedsQl, the calculation of total score intervals was done separately for each age group, including that of their legal guardians^(13)^. To define the categories of QoL levels, firstly, minimum and maximum values were subtracted, respectively, in order to obtain the 1^st^ quartile, i.e., the result obtained divided by 4 (quartiles). This value was then added to establish the intervals according to the very low/low and very high/high QoL classification. For instance, QL levels of children aged 2 to 4 years will be between 51 and 83 and, therefore, we have 83–51 = 32 units. This number was later divided by 4, whose approximate result was 8. Then, in the first quartile, the addition of 51 + 8 = 59 occurred; therefore, the range was between 51 and 59, and so on, until the creation of the 4^th^ quartile so that higher scores indicate better HRQoL.

In the IQVJD, the same procedures were used to obtain the quartiles, but the ranges were established for each domain: satisfaction; impact; concern; and total IQVJD. However, in this instrument, higher scores suggest worse HRQoL.

### Ethical Aspects

The study followed the guidelines of Resolution 466/2012 of the Brazilian National Health Council, which regulates research with human beings in the country. It was approved by the Research Ethics Committee, under Protocol 005327/2020, Opinion 4,454,023. By including the second research setting, subsequent Opinion 4,556,497 was obtained, both approved in 2020 and 2021, respectively. Parents and adolescents participating in the research signed the Informed Consent Form (ICF), and children signed the Informed Assent Form (IAF). Participant anonymity was guaranteed by using codes related to the outpatient centers where each person was monitored, followed by the ordinal number in the sequence of collection.

## RESULTS

Of the 81 (100%) children/adolescents with 1DM and their legal guardians, mothers (n = 73; 90.1%) were the main caregivers. Mothers’ age group ranged between 30 and 39 years (n = 32; 39.5%). Almost half worked as housewives (n = 40; 49.4%), with married marital status (n = 45, 55.6%) and family income of less than a minimum wage (n = 33; 40, 7%). Regarding education, it was found that only 37.3% (n = 25) of their guardians completed high school. Regarding children/adolescents with 1DM, there was a slight predominance of females (n = 43; 53.1%), white (n = 39; 48.1%), age group from 13 to 17 years (n = 33; 40.7%), with the majority enrolled in elementary school (n = 64; 85.3%).

The results related to social and clinical characteristics of guardian caregivers, associated with the HRQoL of children/adolescents with 1DM, were described in Table 1. Adolescents (63.2%) whose parents had a family income greater than a minimum wage (p-value = 0.049) showed a 0.5 times lower prevalence of having a low/very low QoL, when compared to those with a family income lower than a minimum wage.

**Table 1:** Quality of life levels associated with parental characteristics according to the age group of children and adolescents with 1DM, PedsQl – João Pessoa, PB, Brazil, 2021.

Variables	Health–related quality of life		P	PR*	(95%) CI^§^
	Low/very low n(%)	High/very high n(%)			
**Children from 2 to 4 years**					
**Education**					
High school/higher education	4 (57.1)	3 (42.9)	0.500^‡^	0.6	0.3–1.1
Elementary school	2 (100.0)	–		1	–
**Family income**					
>1 minimum wage	2 (28.6)	5 (71.4)	1.000^‡^	1.4	0.3–6.2
≤1 minimum wage	1 (50.0)	1 (50.0)		1	–
**Marital status**					
With a partner	6 (66.7)	3 (33.3)	–	–	–
**Parents have DM**					
No	6 (66.7)	3 (33.3)	–	–	–
**Have children other than child/adolescent with 1DM**					
No	1 (16.7)	5 (83.3)	0.226^‡^	2.5	0.5–12.9
Yes	1 (33.3)	2 (66.7)		1	–
**Children from 5 to 7 years**					
**Education**					
High school/higher education	1 (33.3)	2 (66.7)	0.486^‡^	0.4	0.1–2.4
Elementary school	3 (75.0)	1 (25.0)		1	–
**Family income**					
>1 minimum wage	3 (50.0)	3 (50.0)	1.000^‡^	0.5	0.2–1.1
≤1 minimum wage	1 (100.0)	–		1	–
**Marital status**					
With a partner	4 (66.7)	2 (33.3)	0.429^‡^	–	–
Without a partner	–	1 (100.0)		–	–
**Parents have DM**					
No	2 (50.0)	2 (50.0)	1.000^‡^	0.7	0.2–2.7
Yes	2 (66.7)	1 (33.3)			
**Have children**					
No	1 (50.0)	1 (50.0)	1.000^‡^	0.8	0.2–4.0
Yes	3 (60.0)	2 (40.0)		1	–
**Children from 8 to 12 years**					
**Education**					
High school/higher education	4 (26.7)	11 (73.3)	0.439^†^	0.7	0.2–1.9
Elementary school	6 (40.0)	9 (60.0)		1	–
**Family income**					
>1 minimum wage	5 (31.3)	11 (68.8)	1.000^‡^	0.9	0.3–2.4
≤1 minimum wage	5 (37.5)	9 (64.3)		1	–
**Marital status**					
With a partner	8 (33.3)	16 (66.7)	1.000^‡^	1.0	0.3–3.5
Without a partner	2 (33.3)	4 (66.7)		1	–
**Parents have DM**					
No	8 (33.3)	16 (66.7)	1.000^‡^	1.0	0.3–3.5
Yes	2 (33.3)	4 (66.7)		1	–
**Have children**					
No	5 (45.5)	6 (54.5)	0.425^‡^	1.7	0.6–4.7
Yes	5 (26.3)	14 (73.7)		1	–
**Adolescents from 13 to 18 years**					
**Education**					
High school/higher education	10 (43.5)	13 (56.5)	0.259^‡^	0.6	0.3–1.1
Elementary school	7 (70.0)	3 (30.0)		1	–
**Family income**					
>1 minimum wage	12 (63.2)	7 (36.8)	**0.049** ^†^	**0.5**	**0.3–1.0**
≤1 minimum wage	10 (71.4)	4 (28.6)		1	–
**Marital status**					
With a partner	13 (56.5)	10 (43.5)	0.259^‡^	0.6	0.3–1.1
Without a partner	7 (70.0)	3 (30.0)		1	–
**Parents have DM**					
No	15 (51.7)	14 (48.3)	1.000^‡^	1.0	0.4–2.9
Yes	2 (50.0)	2 (50.0)		1	–
**Have children**					
No	3 (30.0)	7 (70.0)	0.141^‡^	0.5	0.2–1.3
Yes	14 (60.9)	9 (39.1)		1	–

Note: ^†^Pearson’s chi-square; ^‡^Fisher’s exact test; *Prevalence Ratio; ^§^95% Confidence Interval; DM = Diabetes Mellitus; 1DM= Type 1 Diabetes Mellitus.

Table 2 shows that children aged 8 to 12 years who self-administered insulin have a 0.6 times lower prevalence of having a high/very high QoL (p-value = 0.030) compared to those who did not self-administer.

**Table 2:** Quality of life levels associated with sociodemographic and clinical factors of children aged 8 to 12 years according to PedsQl – João Pessoa, PB, Brazil, 2021.

Variables	Health–related quality of life		P	PR*	(95%) CI^§^
	Low/very low n (%)	High/very high n (%)			
**Sex**					
Male	8 (53.3)	7 (46.7)	0.464^†^	1.3	0.1–3.0
Female	6 (40.0)	9 (60.0)		1	–
**Education**					
Elementary school	14 (46.7)	16 (53.3)	–	–	–
**Participates in diabetes education group**					
No	14 (46.7)	16 (53.3)	–	–	–
**Parents have DM**					
No	10 (41.7)	14 (58.3)	0.378^‡^	0.6	0.3–1.3
Yes	4 (66.7)	2 (33.3)		–	–
**Clinical status at 1DM diagnosis**					
Hyperglycemia with signs and symptoms	12 (44.4)	15 (55.6)	–	–	–
Diabetic ketoacidosis	1 (50.0)	1 (50.0)	–	–	–
Other	1 (100.0)	–	–	–	–
**Time since diagnosis**					
Up to 4 years	11 (52.4)	10 (47.6)	0.440^‡^	1.6	0.6–4.3
> 4 years	3 (33.3)	6 (66.7)			
**Result of glycated hemoglobin**					
≤7	3 (42.9)	4 (57.1)	1.000^‡^	1.0	0.3–2.7
>7	7 (43.8)	9 (56.3)		–	–
**Device used to apply basal insulin**					
50 IU insulin syringe	–	1 (100.0)	–	–	–
100 IU insulin syringe	1 (100.0)	–	–	–	–
Insulin syringe with 100 IU needle attached	–	2 (100.0)	–	–	–
Disposable pen	4 (50.0)	4 (50.0)	–	–	–
Rechargeable pen	9 (52.9)	8 (47.1)	–	–	–
Insulin bomb	–	1 (100.0)	–	–	–
**Device used to apply bolus insulin**					
50 IU insulin syringe	–	1 (100.0)	–	–	–
Insulin syringe with 100 IU needle attached	–	2 (100.0)	–	–	–
Disposable pen	10 (50.0)	10 (50.0)	–	–	–
1 IU rechargeable pen	2 (50.0)	2 (50.0)	–	–	–
Insulin bomb	–	1 (100.0)	–	–	–
**Has sites with visible or palpable lipohypertrophy**					
One site	5 (38.5)	8 (61.5)	1.000^‡^	0.8	0.2–2.5
Two sites	2 (50.0)	2 (50.0)	–	–	–
**Self-administers insulin**					
Yes	5 (29.4)	12 (70.6)	**0.030** ^†^	**0.4**	**0.2–1.0**
No	9 (69.2)	4 (30.8)	–	–	–
**Counts carbohydrates**					
Yes	–	3 (100.0)	0.228^‡^	–	–
No	14 (51.9)	13 (48.1)	–	–	–
**Performs physical activity**					
Yes	13 (46.4)	15 (53.6)	1.000^‡^	0.9	0.2–3.9
No	1 (50.0)	1 (50.0)	–	–	–
**How many times has been admitted to hospital due to hypoglycemia**					
None	14 (48.3)	15 (51.7)	1.000^‡^	–	–
Once	–	1 (50.0)	–	–	–
**How many times has been admitted to hospital due to hyperglycemia**					
None	15 (55.6)	12 (44.4)	1.000^‡^	0.9	0.2–3.8
Once	1 (50.0)	1 (50.0)	–	–	–
**How many times has been admitted to hospital due to ketoacidosis**					
None	14 (48.3)	15 (51.7)	1.000^‡^	–	–
Once	–	1 (50.0)	–	–	–

Note: ^†^Pearson’s chi-square; ^‡^Fisher’s exact test; *Prevalence Ratio; ^§^95% Confidence Interval; DM = Diabetes Mellitus; 1DM= Type 1 Diabetes Mellitus.

The results show that adolescents with a diagnosis time of less than 4 years (p-value = 0.009) have a 0.4 times lower prevalence of having low/very low QoL when compared to those with a diagnosis time of more than 4 years (PR = 0.6) for the impact domain (Table 3).

**Table 3:** Quality of life levels in the impact domain associated with sociodemographic and clinical factors of adolescents with 1DM according to IQVJD – João Pessoa, PB, Brazil, 2021.

Variables	Health–related quality of life		P	PR*	(95%) CI^§^
	High/very high n (%)	Low/very low n (%)			
**Sex**					
Male	15 (78.9)	4 (21.1)	0.244^†^	1.3	0.9–1.9
Female	15 (62.5)	9 (37.5)	–	1	–
**Age group**					
12 to 15 years	8 (80.0)	2 (20.0)	–	1	–
16 to 18 years	21 (65.6)	11 (34.4)	0.659^‡^	0.82	0.5–1.2
**Education**					
Elementary school	24 (75.0)	8 (25.0)			
High school	6 (60.0)	4 (40.0)	1.000^‡^	1.5	0.4–6.3
Higher education	–	1 (100.0)			
**Participates in diabetes education group**					
Yes	1(100.0)	–	–	1	–
No	29 (69.0)	13 (31.0)	1.000^‡^	1.4	1.2–1.8
**Parents have DM**					
No	27 (75.0)	9 (25.0)	–	1	–
Yes	3 (50.0)	3 (50.0)	0.329^‡^	1.5	0.7–3.4
**Clinical status at 1DM diagnosis**					
Hyperglycemia with signs and symptoms	24 (70.6)	10 (29.4)			
Diabetic ketoacidosis	4 (57.1)	3 (42.9)			
Asymptomatic	2 (100.0)	–			
**Time since diagnosis**					
Up to 4 years	8 (47.1)	9 (52.9)	**0.009** ^†^	**0.6**	**0.3–0.9**
> 4 years	22 (84.6)	4 (15.4)	–	1	–
**Result of glycated hemoglobin**					
≤7	6 (85.7)	1 (14.3)	1.000^‡^	1.1	0.8–1.6
>7	22 (78.6)	6 (21.4)	–	1	–
**Device used to apply basal insulin**					
50 IU insulin syringe	1 (100.0)	–			
100 IU insulin syringe	–	3 (100.0)			
Insulin syringe with 100 IU needle attached	–	3 (100.0)			
Disposable pen	9 (75.0)	3 (25.0)			
Rechargeable pen	19 (82.6)	4 (17.4)			
Insulin bomb	1 (100.0)	–			
**Device used to apply bolus insulin**					
50 IU insulin syringe	1 (100.0)	–			
100 IU insulin syringe	–	1 (100.0)			
Insulin syringe with 100 IU needle attached	1 (33.3)	2 (66.7)			
Disposable pen	23 (74.2)	8 (25.8)			
1 IU rechargeable pen	4 (100.0)	–			
0.5 IU rechargeable pen	–	1 (100.0)			
Insulin bomb	1 (100.0)	–			
**Has sites with visible or palpable lipohypertrophy**					
One site	11 (61.1)	7 (38.9)	–	1	–
Two sites	3 (75.0)	1 (25.0)	0.999^‡^	1.2	0.6–2.4
**Self-administers insulin**					
Yes	28 (70.0)	12 (30.0)	1.000^‡^	1.1	0.5–2.4
No	2 (66.7)	1 (33.3)	–	1	–
**Counts carbohydrates**					
Yes	6 (85.7)	1 (14.3)	–	1	–
No	24 (66.7)	12 (33.3)	0.412^†^	1.3	0.9–1.9
**Performs physical activity**					
Yes	27 (69.2)	12 (30.8)	–	1	–
No	3 (75.0)	1 (25.0)	1.000^‡^	1.2	0.1–7.2
**How many times has been admitted to hospital due to hypoglycemia**					
None	29 (69.0)	13 (31.0)	–	1	–
Once	1 (100.0)	–	1.000^‡^	0.7	0.6–0.8
**How many times has been admitted to hospital due to hyperglycemia**					
None	28 (71.8)	11 (28.2)	–	1	–
Once	2 (50.0)	2 (50.0)	0.572^‡^	1.4	0.5–3.9
**How many times has been admitted to hospital due to ketoacidosis**					
None	29 (70.7)	12 (29.3)	–	1	–
Once	1 (50.0)	1 (50.0)	0.518^‡^	1.4	0.3–5.7

Note: ^†^Pearson’s chi-square; ^‡^Fisher’s exact test; *Prevalence Ratio; ^§^ 95% Confidence Interval; DM = Diabetes Mellitus; 1DM= Type 1 Diabetes Mellitus.

## DISCUSSION

This study analyzed clinical and sociodemographic factors associated with HRQoL of children and adolescents with 1DM. From the profile design, sociodemographic and clinical characteristics of children/adolescents with 1DM and their guardians were identified, which are intrinsically related to disease management.

When assessing social factors of caregivers on the HRQoL of children/adolescents with 1DM, a significant association was found between a higher level of HRQoL and parents with family income greater than a minimum wage, i.e., there was a lower prevalence of having compromised HRQoL compared to those with income below a minimum wage. Another research also identified an association between low income and ineffective metabolic control and worse HRQoL, considering that adolescents with 1DM belonging to more underprivileged socioeconomic strata may be more susceptible to complications. This fact contributes to reducing the life expectancy and HRQoL of these young people, as indicated by data analysis^(15)^.

Children aged 8 to 12 years are recognized for their nuances and singularities, and these, in the case of 1DM, permeate changes that involve insulin sensitivity, physical growth, the ability to initiate self-care and even neurological vulnerability to hypoglycemia, which may interfere with 1DM self-management and HRQoL. Study showed a statistically significant association between HRQoL and self-administration of insulin, biochemical results, number of hospital admissions and the presence of complications^(8)^.

In the concern domain, male adolescents showed a high level of HRQoL. In line with this finding, a study revealed that the gender variable is directly associated with the QoL of young people with 1DM and that boys reported a more satisfactory HRQoL than girls^(8)^. This association has been the subject of discussions due to the way of coping in 1DM management.

Based on other research, girls with 1DM had a 4.5 times higher prevalence of developing psychological distress when compared to their male peers^(14)^. This condition is linked to the way they deal with the demands of 1DM, which can have an impact on HRQoL. Previous studies corroborate this evidence and highlight that this propensity is even more common in adolescence, due to increased worries, lower satisfaction with life and negative perception of their health status^(15,16)^. The biological nature of 1DM is another factor that leads to the emergence of psychological disorders, given the severe fluctuations in glucose, which impair the functioning of the central nervous system, responsible for cognition and mood^(8,17)^.

Time since diagnosis of 1DM is another equally important factor, capable of affecting adolescents’ QoL. Therefore, children and adolescents with DM diagnosed more than five years ago are more likely to have a worse HRQoL due to diabetes distress, triggered by long-term coexistence with the disease and the psychosocial impact^(8)^. This may be explained by prolonged exposure to 1DM management stresses and a higher rate of complications. Studies carried out in Egypt, Poland and the United States confirm this association^(16,17)^.

The aforementioned results confirm the originality of this study due to the cultural and regional diversity present in the country and contribution to the advancement of knowledge necessary to support the educational process in diabetes. Health education actions must consider the unique needs of these people at each step of the disease^(18, 19, 20)^. It is important to highlight that the data obtained are relevant, as it presents the association of these factors in the HRQoL of children/adolescents with 1DM and HRQoL assessment through two specific instruments validated nationally.

As limitations of this investigation, small sample size, as it reflected in evidence of statistically significant associations, and difficulties during remote collection stand out, due to the fact that the respondent fills out the instruments without the researcher’s supervision, a condition that can lead to risk of bias. Despite this, the results presented provide support for redirecting health decision-making, focusing on multidisciplinary care beyond the biological dimension of care.

## CONCLUSION

In this study, it was found that the clinical and sociodemographic factors associated with the HRQoL of children and adolescents with 1DM were minimum wage, time since diagnosis and self-administration of insulin. Young people whose parents had an income below a minimum wage had a higher prevalence of having low/very low QoL. The importance of financial support for low-income families is highlighted, given its negative influence on HRQoL.

In the isolated analysis of the IQVJD impact domain, a significant association was found with time since diagnosis of up to 4 years as a protective factor for low/very low HRQoL level. Therefore, greater attention should be paid to adolescents with more than 4 years of diagnosis, due to the lower prevalence of high/very high HRQoL levels.

Concerning 1DM management, cases of self-administration of insulin by children between 8 and 12 years old revealed a lower prevalence of having high/very high QoL compared to those who did not self-administer, according to PedsQl. In this age group, in which there is generally a transition from parents to children, attention is drawn to the importance of expanding support from the social network, the multidisciplinary team and the family, in order to minimize the unfavorable repercussions on HRQoL.

The particularities of each age group, individual characteristics and social context are important factors that must be taken into account when monitoring this audience. Understanding the repercussions of 1DM on children’s and adolescents’ HRQoL makes it feasible to develop public policies that favor timely access to health, guarantee continuity of care and improvements in quality of care.
